# Supplementary material for: HIVprotI: an integrated web based platform for prediction and design of HIV proteins inhibitors
Source: J Cheminform. 2018 Mar 9;10:12. doi: 10.1186/s13321-018-0266-y (PMC5845081; doi:10.1186/s13321-018-0266-y)
Supplement: Supplementary file 2 — Additional file 2. Source code of HIVProtI web server. [file 13321_2018_266_MOESM2_ESM.zip › HIVprotI_Source-code/hivprotI/showmain_batch.php]

php error\_reporting(0);
include("head.php");?

php
#####pasted file#####
$str=$\_POST['str'];
##########uploaded file####
$filename = basename($\_FILES['file']['name']);
$filename = trim("$filename");
if (is\_uploaded\_file($\_FILES['file']['tmp\_name']))
$str1= file\_get\_contents($\_FILES['file']['tmp\_name']);
#################
$email = $\_POST["email"];
$ic = $\_POST["ic"];
if($ic==''){$ic="0";}
########### form values ########
$int = $\_REQUEST['int'];
$pr = $\_REQUEST['pr'];
$rt = $\_REQUEST['rt'];
#############################
$ran=rand(100,100000);
//echo $ran."<br/";
$dir="/home/gpsr/webserver/cgidocs/tmp/manojk/hivproti/first\_$ran";
exec("mkdir $dir");
exec("chmod 777 $dir");
exec("chmod 777 $dir/\*");
$FP1=fopen("$dir/input.sdf","w");
if($str != ""){
fwrite($FP1,$str);
}
else{$FP1=fopen("$dir/input.sdf","w");
fwrite($FP1,$str1);}
fclose($FP1);
if(($str=="") && ($str1=="")){echo"

### Please either paste your structure or upload file!!

";}
elseif(($str!="") && ($str1!="")){echo"

### Please either paste your structure or upload file, not both!!

";}
else{
#########model selection########
$FP2=fopen("$dir/model\_req","w");
if($int != ""){fwrite($FP2,"$int,");}
if($pr != ""){fwrite($FP2,"$pr,");}
if($rt != ""){fwrite($FP2,"$rt,");}
#if($hupt3 != ""){fwrite($FP2,"$hupt3,");}if($hupt4 != ""){fwrite($FP2,"$hupt4,");}if($kp4 != ""){fwrite($FP2,"$kp4,");}if($mia != ""){fwrite($FP2,"$mia,");}if($mz1 != ""){fwrite($FP2,"$mz1,");}if($panc3 != ""){fwrite($FP2,"$panc3,");}if($panc8 != ""){fwrite($FP2,"$panc8,");}if($panc10 != ""){fwrite($FP2,"$panc10,");}if($psn1 != ""){fwrite($FP2,"$psn1,");}if($sw != ""){fwrite($FP2,"$sw,");}if($yapc != ""){fwrite($FP2,"$yapc,");}
fclose($FP2);
exec("perl -pi -e 's/,$//g' $dir/model\_req");
exec("perl -pi -e 's/,/\n/g' $dir/model\_req");
//$models=file\_get\_contents("$dir/model\_req");
//echo"$models  
";
################################
####### main programm ########
#system("php /home/gpsr/webserver/cgidocs/manojk/hivproti/sub\_batch.php $ran $ic >/dev/null &");
##############################
exec("perl -pi -e 's/\r//g' $dir/input.sdf");
exec("perl /home/gpsr/webserver/cgidocs/manojk/hivproti/single-sdf.pl $dir/input.sdf $ran");
#exec("rm $dir/input.sdf");
exec("ls $dir/\*.sdf |grep -v 'input' >$dir/list\_sdf");
exec("perl /home/gpsr/webserver/cgidocs/manojk/hivproti/energy\_min.pl $ran");
exec("mkdir $dir/min\_str");
exec("mv $dir/\*\_min.sdf $dir/min\_str");
#exec("rm $dir/\*.sdf");
shell\_exec("java -jar /home/gpsr/webserver/cgidocs/manojk/hivproti/padel/PaDEL-Descriptor.jar -2d -3d -fingerprints -descriptortypes /home/gpsr/webserver/cgidocs/manojk/hivproti/descriptors.xml -dir $dir/min\_str -file $dir/desc >/dev/null");
exec("perl -pi -e 's/,,/,0,/g' $dir/desc");
exec("perl -pi -e 's/,,/,0,/g' $dir/desc");
exec("perl /home/gpsr/webserver/cgidocs/manojk/hivproti/desc.pl $dir/desc $ran");
exec("ls $dir/desc\_ana\_\* > $dir/list");
exec("perl /home/gpsr/webserver/cgidocs/manojk/hivproti/for\_sever1.pl $ran /home/gpsr/webserver/cgidocs/manojk/hivproti/arff/list1\_arff");
#exec("ls $dir/General/\*.arff >$dir/General\_list");
exec("ls $dir/RT/\*.arff >$dir/RT\_list");
exec("ls $dir/PR/\*.arff >$dir/PR\_list");
exec("ls $dir/INT/\*.arff >$dir/INT\_list");
#exec("ls $dir/HIV/\*.arff >$dir/HIV\_list");
exec("perl /home/gpsr/webserver/cgidocs/manojk/hivproti/knn3\_model.pl /home/gpsr/webserver/cgidocs/manojk/hivproti/arff/list2\_arff $ran");
#exec("more $dir/General/\*\_acc >$dir/General\_acc");
exec("more $dir/RT/\*\_acc >$dir/RT\_acc");
exec("more $dir/PR/\*\_acc >$dir/PR\_acc");
exec("more $dir/INT/\*\_acc >$dir/INT\_acc");
exec("perl -pi -e 's/\//\t/g' $dir/list\_sdf");
#exec("cut -f8 $dir/list\_sdf >$dir/ids");
#exec("perl -pi -e 's/\.sdf//g' $dir/ids");
####### desc calculation (filterit)######
exec("perl /home/gpsr/webserver/cgidocs/manojk/hivproti/filter\_batch.pl $ran");
exec("perl /home/gpsr/webserver/cgidocs/manojk/hivproti/desc\_filter\_batch.pl $ran");
#########################################
exec("cut -f1 $dir/desc\_filter1 > $dir/ids");
exec("perl /home/gpsr/webserver/cgidocs/manojk/hivproti/process\_acc\_batch.pl $ran $ic");
exec("perl /home/gpsr/webserver/cgidocs/manojk/hivproti/make\_topkey.pl $ran");
exec("cat $dir/topkey $dir/final\_acc bottom > $dir/cat\_final\_accuracy");
exec("cat $dir/topkey\_d $dir/final\_acc\_download > $dir/cat\_final\_accuracy\_d");
exec("rm -r $dir/RT $dir/PR $dir/INT");
##############################
##############################
if($email==""){
echo "";}
else{
echo "";}
}
?>

php include "foot.php"?
